# Integrated multi-biomarker panel of CXCL13, HS-CRP, and WBC counts predicts outcomes in stroke neurosyphilis patients treated with HBO and TUS-NMES

**DOI:** 10.3389/fneur.2025.1530447

**Published:** 2025-02-25

**Authors:** Wenchao He, Shuangshuang Chen, Ruyang Chen, Jun Zhang, Xuehua Zhang, Minzhi Wu, Dan Zhang, Fengfeng Zhu, Fanghua He, Yating Xv, Na Lei, Wenhui Zheng, Xinyi Shan, Jun Dai

**Affiliations:** ^1^Department of Rehabilitation, The Fifth People’s Hospital of Suzhou, The Affiliated Infectious Disease Hospital, Suzhou Medical College of Soochow University, Suzhou, China; ^2^Hospital Department, The Fifth People’s Hospital of Suzhou, The Affiliated Infectious Disease Hospital, Suzhou Medical College of Soochow University, Suzhou, China; ^3^Department of Dermatology, The Fifth People’s Hospital of Suzhou, The Affiliated Infectious Disease Hospital, Suzhou Medical College of Soochow University, Suzhou, China; ^4^Intensive Care Unit, The Fifth People’s Hospital of Suzhou, The Affiliated Infectious Disease Hospital, Suzhou Medical College of Soochow University, Suzhou, China; ^5^Clinical Laboratory, The Fifth People’s Hospital of Suzhou, The Affiliated Infectious Disease Hospital, Suzhou Medical College of Soochow University, Suzhou, China

**Keywords:** stroke neurosyphilis, hyperbaric oxygen, transcranial ultrasound neuromuscular stimulation, C-X-C motif chemokine ligand 13, biomarkers

## Abstract

**Background:**

Neurosyphilis results from *Treponema pallidum* invading the central nervous system, leading to severe neurological issues like stroke. Combining hyperbaric oxygen (HBO) therapy and transcranial ultrasound neuromuscular stimulation (TUS-NMES) shows promise in improving outcomes.

**Objective:**

This study evaluates the predictive value and clinical significance of CXCL13, WBC, and Hs-CRP levels in neurosyphilis patients undergoing HBO and TUS-NMES therapy.

**Methods:**

The study included 158 neurosyphilis stroke patients treated from June 2022 to January 2024. Assessments of limb motor, cognitive functions, daily living abilities, and cerebrospinal fluid biomarkers were conducted pre- and post-four weeks of combined therapy.

**Results:**

After treatment, there was a significant improvement in FMA, MoCA, and MBI scores (*p* < 0.001). CXCL13 levels significantly decreased post-treatment, correlating with improved patient outcomes. The study found strong predictive values for CXCL13 levels in determining the efficacy of rehabilitation, with the combination of CXCL13, WBC, and Hs-CRP showing the highest predictive accuracy.

**Conclusion:**

HBO and TUS-NMES significantly enhance recovery in neurosyphilis stroke patients. CXCL13, WBC, and Hs-CRP effectively predict rehabilitation outcomes, highlighting their value in clinical management.

## Introduction

Syphilis, classified as a serious Class B infectious disease in China, can damage vital organs and significantly impact health. Over 20% of early-stage patients who are untreated or inadequately treated may develop neurosyphilis, as *Treponema pallidum* invades the central nervous system. This can occur early in the infection or within a few years. Neurosyphilis can lead to syphilitic vascular damage, resulting in thrombosis, strokes, and neurological impairments that affect limb function and reduce the quality of life (1, 2).

Consequently, clinical research has increasingly focused on restoring central nervous system functions, improving limb dysfunction, and enhancing the daily life quality of patients, especially following the timely and adequate eradication of *Treponema pallidum* with antisyphilitic medications (3). This treatment approach involves not only the effective removal of the pathogen but also addressing the residual effects on the nervous system and motor functions. Hyperbaric oxygen therapy (HBO) is known to improve tissue hypoxia by supplying a high concentration of oxygen, which aids in neovascularization and the repair of brain microcirculation (4). While many clinical studies have confirmed that HBO can help recover central nervous system functions and improve neurological outcomes in stroke patients, its impact on limb dysfunction remains minimal (5, 6).

To maximize rehabilitation outcomes, current guidelines recommend a multifaceted approach involving various combined treatment modalities. This strategy has been shown to enhance the effects of rehabilitation for stroke-related sequelae and achieve better results. Transcranial ultrasound neuromuscular stimulation (TUS-NMES) represents a novel technology that incorporates both ultrasound and neuromuscular electrical stimulation. It simultaneously stimulates the central and peripheral nerves, enhancing limb function control (7, 8). It is hypothesized that combining these therapies could improve the rehabilitation of stroke neurosyphilis patients from various angles, thereby enhancing treatment efficacy.

However, the assessment of therapeutic outcomes in stroke neurosyphilis rehabilitation primarily relies on patient-centered subjective measures such as scales, and there remains a lack of objective, specific indicators. The CXC chemokine family, particularly the motif chemokine ligand 13 (CXCL13), plays a critical role in controlling B-cell aggregation and triggering aberrant humoral immune responses. Its abnormal expression in cerebrospinal fluid can indicate the severity of neurosyphilis and provide valuable guidance for treatment strategies. Additionally, an increase in the white blood cell count in the cerebrospinal fluid is a crucial diagnostic marker of neurosyphilis, reflecting the inflammation within the central nervous system. High-sensitivity C-reactive protein (Hs-CRP) is also a key indicator of systemic inflammation, and variations in its levels can signal disease activity or the presence of other concurrent inflammatory conditions.

Despite extensive research on the efficacy, trends, and clinical implications of combining HBO with TUS-NMES for treating stroke neurosyphilis, significant variations in trial designs and observational metrics have prevented reaching a uniform and convincing conclusion. Therefore, further research is essential to demonstrate the therapeutic value of this approach more conclusively and to evaluate the predictive value of changes in clinical and laboratory indices on patient rehabilitation outcomes. This study was specifically conducted by our hospital to address these gaps.

## Patients and methods

This prospective study aimed to evaluate the predictive value and clinical significance of CXCL13, WBC, and Hs-CRP levels in neurosyphilis patients undergoing HBO and TUS-NMES therapy. Participants were recruited from Suzhou No. 5 People’s Hospital, Soochow University Affiliated Infectious Disease Hospital. The recruitment process involved initial screenings followed by detailed assessments to confirm eligibility, which are documented to enable precise replication. This study was approved by the ethical committee of the fifth People’s Hospital of Suzhou, The Affiliated Infectious Disease Hospital, Suzhou Medical College of Soochow University (SZWY20230913-03), and was conducted in compliance with Declaration of Helsinki. Informed consent was obtained from all participants.

### Sample size calculation

Our sample size was calculated to ensure robust statistical power for detecting a clinically significant difference in serum CXCL13 levels following the therapy. The calculation is based on the standard formula for determining the sample size needed to compare two independent means:


$$n=2\left(\frac{{\left(Z_{\frac{\alpha }{2}+}Z_{\beta }\right)}^{2}X\;(\sigma_{1}^{2}+\sigma_{2}^{2}}{\delta^{2}}\right)$$

Where:

- Z_*α*/2_ is the critical value of the normal distribution at α/2 (for a two-tailed test at α = 0.05, Z_α/2_ = 1.96).
- Z*_β_* is the critical value of the normal distribution at β (for *β* = 0.1, Z_β_ = 1.282).
- σ1 and σ2 are the standard deviations of serum CXCL13 levels before and after treatment, respectively (8.04 and 7.82).
- *δ* is the difference in means between the two groups, targeted at 7.11, based on preliminary studies.

Using these parameters, the formula calculates a sample size of approximately 128 patients. To account for a potential dropout rate of 10%, we adjusted the total number required to approximately 141. To ensure an even more conservative approach and enhance the study’s power, we ultimately included 158 patients in the study.

This study enrolled 158 stroke neurosyphilis patients treated at our institution from June 2022 to January 2024. The inclusion criteria were: (1) confirmation of neurosyphilis diagnosis as previous described (9); (2) positive serum rapid plasma regain (RPR) test for *Treponema pallidum*; (3) acute presentation with focal neurological deficits confirmed by MRI as acute ischemic stroke (10); (4) first episode of limb dysfunction; (5) negative penicillin skin test; (6) no contraindications to the rehabilitation treatments employed in this study. The exclusion criteria included: (1) recent treatment with antiviral agents, interferon, immunosuppressants, or hormones within the past 30 days; (2) prior antisyphilitic treatment; (3) presence of systemic immune diseases such as systemic lupus erythematosus or rheumatic diseases; (4) major organ dysfunction or malignant tumors; (5) women who were pregnant or lactating; (6) cognitive or mental disorders; (7) other concurrent infections like AIDS; (8) blood transfusion within the last 30 days; (9) re-infection with syphilis after enrollment or during follow-up. The study was authorized by the Medical Ethics Committee of our hospital, and informed consent was obtained from all participants.

### Data collection

Data were collected at baseline, immediately after the intervention, and at 1-month follow-up. Clinical outcome measures included motor function, cognitive function, and daily living activities, assessed using standardized scales such as the Fugl-Meyer Assessment, Montreal Cognitive Assessment, and Barthel Index. Data collection methods were standardized through training sessions and calibration exercises to ensure consistency.

Upon enrollment, all patients underwent a combined treatment regimen including routine symptomatic care, hyperbaric oxygen (HBO), and transcranial ultrasound neuromuscular electrical stimulation (TUS-NMES).

### Penicillin treatment for neurosyphilis

− Initial phase: Penicillin 4 million U intravenous drip administered over 4 h per day for 14 consecutive days.
− Follow-up phase: Benzylpenicillin 2.4 million U given as an intramuscular injection once weekly for 3 weeks.

### Basic stroke management

− Included standard anti-platelet therapy (aspirin, 100 mg, daily). Clopidogrel (75 mg, daily) was added for patients with a history of aspirin intolerance or in cases of recurrent vascular events despite aspirin therapy. Regarding anti-arteriosclerosis measures, all participants received atorvastatin 20 mg daily to manage cholesterol levels.
− Management of comorbid conditions such as hypertension and diabetes.
− Routine limb exercises and other rehabilitation training to enhance daily self-care capabilities were conducted for 90 min daily, 5 days a week, for 4 weeks.

### HBO protocol

− Treatment was administered in a medical air pressurized oxygen chamber manufactured by Shanghai Baobang Medical Equipment Co., Ltd.
− The chamber’s pressure was increased to 2 atmospheres, and patients inhaled oxygen at this pressure for 60 min, followed by a 20-min decompression period.
− Administered daily, 5 days a week, for 4 weeks.
− Patients experiencing ear tightness or pain were advised to drink more water or perform saliva swallowing actions. If discomfort persisted, the treatment was paused or the pressure was decreased.

Of the 158 enrolled participants, 139 (88.0%) successfully completed the full course of standard HBO treatments at 2 ATA for 60 min daily, 5 days per week, for four consecutive weeks. The remaining 19 (12.0%) patients experienced varying degrees of discomfort—primarily ear or sinus pressure-related—that led to modifications in their treatment: 12 patients (7.6% of the total) required a reduced operating pressure of approximately 1.8 ATA to manage persistent ear or sinus barotrauma symptoms. These patients completed the same total number of sessions (20 sessions over 4 weeks) but at the lower pressure. 7 patients (4.4% of the total) had incomplete HBO treatment courses due to repeated discomfort or other logistical barriers. Specifically, 4 missed or discontinued >90% of their scheduled HBO sessions, 2 missed or discontinued >75% but <90%, and 1 missed or discontinued >50% but <75%.

### TUS-NMES protocol and electrode placement

All patients underwent TUS-NMES once daily, 5 days per week, for four consecutive weeks. For the neuromuscular electrical stimulation component, electrodes were primarily applied to the affected limbs to target specific muscle groups that were weakened or paretic due to stroke. The standard practice was to place surface electrodes over the motor points of the major muscle groups involved in functional movements. For upper limb deficits, these commonly included the biceps brachii (for elbow flexion), wrist extensors (for wrist and finger extension), and triceps brachii (for elbow extension). For lower limb involvement, electrodes were placed over the quadriceps (for knee extension), tibialis anterior (for ankle dorsiflexion), and gastrocnemius or soleus (for ankle plantarflexion), as indicated by individual patient assessments.

In cases of bilateral involvement, the electrode placement was bilateral but asymmetrical, focusing on the side with more pronounced weakness while providing low-intensity stimulation to the contralateral limb when mild deficits were present. If the patient exhibited near-normal function on the contralateral side, we did not apply electrodes there. Conversely, when patients had bilateral but relatively symmetrical deficits, symmetrical electrode placement was considered to ensure both sides received appropriate stimulation.

Electrode placement decisions were made by a certified rehabilitation physician and physical therapist, based on each patient’s clinical presentation, muscle strength grades, and functional goals. Before therapy initiation, the team performed a brief manual muscle test and palpation to identify ideal motor points. Electrode locations were marked on the skin to maintain consistency for each treatment session. During stimulation, the current amplitude was gradually increased until visible muscle contraction was observed without causing discomfort to the patient.

− Performed using a device from Taizhou Huian Medical Technology Co., Ltd.
− Patients were positioned supine with two ultrasound heads placed in the bilateral temporal windows and one at the stroke focus.
− Hair at the site was removed, a coupling agent applied, and an elastic headband used to maintain close contact.
− The ultrasound duty cycle was set at 60%, with a maximum output power of 0.54 W and intensity of 0.27 W/cm^2^.
− Electrodes for electrical stimulation were positioned on various muscle groups including the upper limb flexors, lower limb popliteal chorda, tibialis anterior, and both long and short fibula muscles.
− Treatment settings included a pulse width of 100 μs and a frequency of 300 Hz, with intensity adjusted to patient tolerance.
− Treatments lasted 30 min, administered once daily, 5 days a week for 4 weeks.
− This rigorous and multifaceted treatment approach aimed to address both the underlying syphilitic infection and the neurological and functional impairments resulting from stroke, using a blend of traditional and innovative therapies to maximize patient recovery.

### Observation index

The assessment protocols and outcome measures used in this study on stroke neurosyphilis patients treated with routine symptomatic treatment and HBO combined with TUS-NMES were comprehensive, encompassing motor and cognitive functions, self-care abilities, and specific biochemical markers:

#### Limb motor function

The Fugl-Meyer Assessment (FMA) (11) was utilized to evaluate limb motor function both before treatment and 4weeks afterward. This assessment covered a total of 33 items, with 16 items (66 points) dedicated to upper limb movement and 17 items (34 points) for lower limb movement, making a total score of 100. A score of 96 or higher was indicative of mild motor dysfunction, with higher scores reflecting less severe dysfunction.

#### Cognitive function

Cognitive function was assessed using the Montreal Cognitive Assessment (MoCA) scale (12), which evaluates various cognitive domains such as executive ability, naming, attention, language skills, delayed recall, orientation, and abstract ability. Notably, memory was not included in the scoring. Assessments were conducted both pre-treatment and 4 weeks post-treatment, with a full score of 30 where a score below 26 indicated mild cognitive impairment.

#### Self-care ability of daily living

The Modified Barthel Index (MBI) (13) was used to measure functional capabilities related to daily living activities such as mobility, eating, stair navigation, and bed-chair transfers. Assessments were made at baseline and after 4 weeks of treatment. A total score of 60 or less suggested a minor handicap, with the scale ranging from 0 to 100 points.

#### Cerebrospinal fluid analysis

Approximately 3 mL of CSF was collected via lumbar puncture at admission and 4 weeks post-treatment, processed by centrifugation, and analyzed for CXCL13 levels using ELISA, and white blood cell count using a Sysmex XE-5000 analyzer. Hs-CRP levels were also measured using a kit from Beijing Baiaolaibo Technology Co., Ltd.

### Efficacy evaluation

− Clinical efficacy was categorized as follows:
− Highly effective: Serum RPR titer was less than 1, with an MBI score of 96 or higher.
− Effective: Serum RPR titer decreased from less than 1 at admission to 95 or less at discharge, with functional independence in daily activities.
− Ineffective: No change or increase in serum RPR titer, no improvement in limb function, and continued dependence on others for daily activities.

$$\begin{array}{l} \mathrm{The total effectiveness rate was calculated}\;as\\ \;\left(\begin{array}{l} \mathrm{number of effective}\;\\ \mathrm{cases}+\mathrm{highly effective cases} \end{array}\right)/\mathrm{total cases}\times 100\%\text{.} \end{array}$$

### Predictive value of CXCL13

The study also aimed to determine the predictive value of CXCL13 levels in CSF for the rehabilitation outcomes of patients. Based on the efficacy evaluation results, patients were categorized into effective and ineffective treatment groups. A receiver operating characteristic curve (ROC) was used to analyze the predictive capability of CXCL13 levels on the effectiveness of rehabilitation therapy, comparing clinical data, FMA, MoCA, MBI scores, and CSF CXCL13 levels pre-treatment between the two groups.

This structured approach ensures a thorough evaluation of treatment impacts on various functional and biological parameters, aiding in a comprehensive understanding of therapy effectiveness in stroke neurosyphilis rehabilitation.

### Statistical analysis

**Figure 1:**
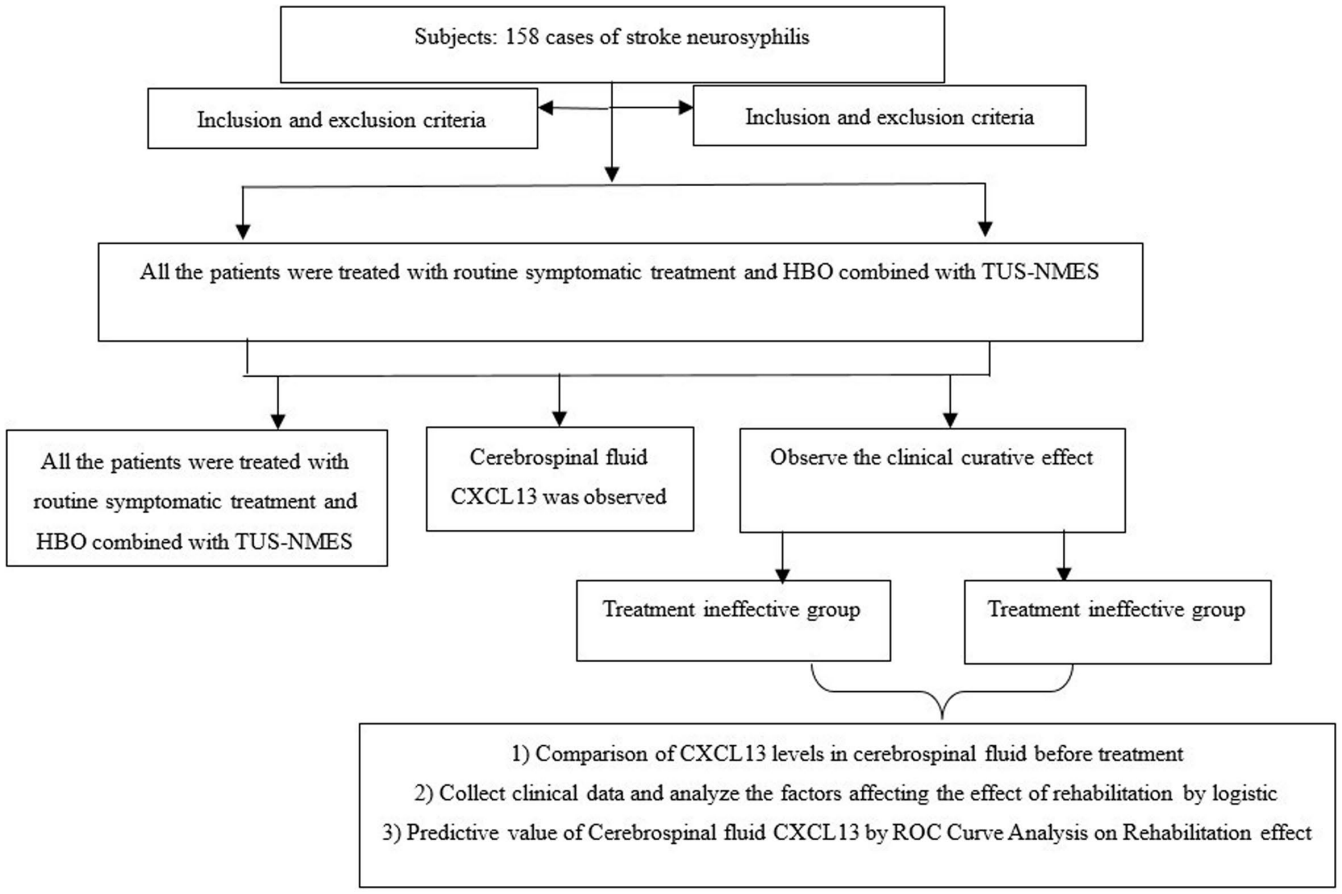
Research flow chart.

Statistical evaluations were performed using SPSS version 23.0 (IBM, Armonk, NY, USA) and graphical representations were processed with GraphPad Prism version 9.4.1. Data were initially tested for normality using the Shapiro–Wilk test and for homogeneity of variances using Levene’s test to ensure the appropriateness of parametric tests. Measurement data, confirmed to follow a normal distribution and with uniform variance, were expressed as mean ± standard deviation (SD). For comparative analyses, the independent sample t-test was employed for assessing inter-group differences, and the paired t-test was used for intra-group comparisons pre- and post-intervention. Categorical data were analyzed using the χ^2^ test, with results presented as counts and percentages. To further explore the factors influencing the rehabilitation outcomes in stroke neurosyphilis patients, logistic regression analysis was performed. This included calculating odds ratios (OR) with 95% confidence intervals (CI) for variables that were significant in univariate analyses. Receiver Operating Characteristic (ROC) curve analysis was utilized to evaluate the predictive power of the biomarkers (CXCL13, WBC, and Hs-CRP). The area under the ROC curve (AUC) was calculated to assess the discriminatory ability of single biomarkers and their combinations, with AUC values interpreted as follows: below 0.5 indicated no discrimination, 0.5–0.69 was considered poor, 0.7–0.89 indicated moderate discrimination, and 0.9–1.0 suggested excellent predictive power. Each test was two-tailed, and a *p*-value of less than 0.05 was considered statistically significant (Figure 1).

## Results

### Characteristics of participants

The study involved 158 patients diagnosed with stroke neurosyphilis, treated at our institution. The clinical characteristics of participants were shown in Table 1.

**Table 1:** Baseline characteristic of participants.

Characteristic	Value
Gender, *n* (%)	
Female	45 (28.5%)
Male	113 (71.5%)
Age (years), Mean ± SD	51.12 ± 4.39
BMI (kg/m^2^)	22.56 ± 2.58
Location of lesion	
Frontal lobe	62 (39.2%)
Temporal lobe	54 (34.2%)
Limb dysfunction	
Left	68 (43.0%)
Right	79 (50.0%)
Bilateral	11 (7.0%)
Motor weakness (paresis or paralysis)	145 (91.8%)
Sensory impairments	49 (31.0%)
Ataxia or coordination deficits	21 (13.3%)
Combined deficits	45 (28.2%)
Presenting symptoms	
Motor weakness	158 (100%)
Impaired speech	102 (64.6%)
Sensory deficits	88 (55.7%)
Comorbidities	
Hypertension	40 (25.3%)
Diabetes mellitus	42 (26.6%)
Cardiovascular diseases	37 (23.4%)
Medications	
Penicillin therapy	158 (100%)
Antiplatelet agents	116 (73.4%)
Statins	59 (37.3%)
Antihypertensive medications	81 (51.3%)
FMA score	79.19 ± 5.39
MoCA score	19.19 ± 2.38
MBI score	65.18 ± 5.87

### Comparison of clinical data of patients with neurosyphilis after stroke before and after treatment

After 4 weeks of treatment, the scores of FMA, MoCA and MBI were significantly higher than those before treatment (*p* < 0.001) (Table 2).

**Table 2:** Comparison of FMA, MoCA and MBI before and after treatment in patients with stroke neurosyphilis.

	Before treatment(*n* = 158)	After 4 weeks of treatment(*n* = 158)	t	*P*
FMA	79.19 ± 5.39	90.21 ± 8.45	13.82	<0.001
MoCA	19.19 ± 2.38	24.41 ± 3.45	15.65	<0.001
MBI	65.18 ± 5.87	80.14 ± 7.91	19.09	<0.001

### Comparison of CXCL13, WBC, Hs-CRP in cerebrospinal fluid of stroke patients with neurosyphilis before and after treatment

**Figure 2:**
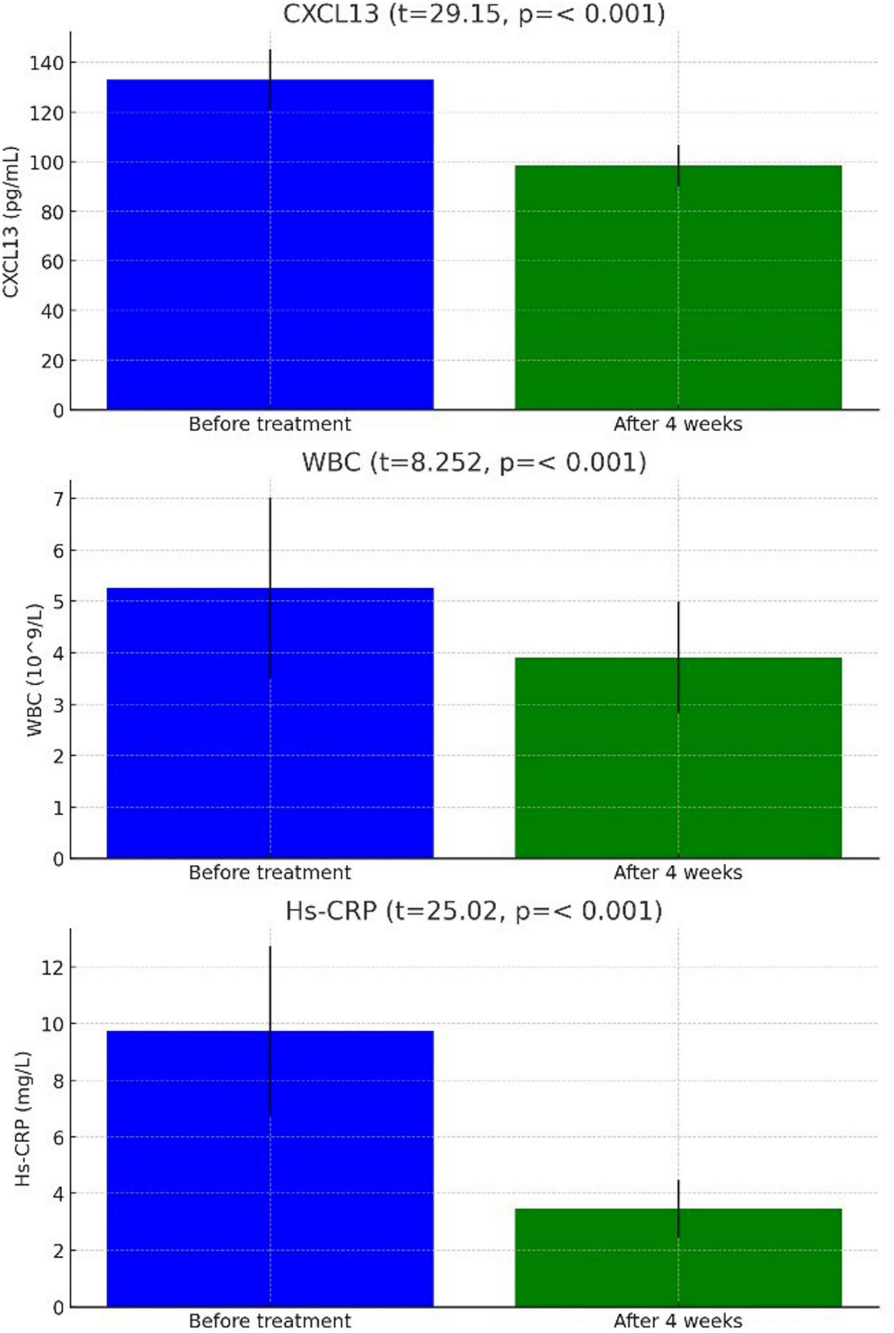
Comparison of CXCL13, WBC, Hs-CRP in cerebrospinal fluid of stroke patients with neurosyphilis before and after treatment.

The CXCL13 level before treatment was (132.91 ± 12.31) μg/mL, which decreased to (98.39 ± 8.37) μg/mL after 4 weeks of treatment, showing a highly significant difference between the two (*t* = 29.15, *p* < 0.001). The WBC count before treatment was (5.26 ± 1.75)*10^9^/L, which decreased to (3.91 ± 1.08)*10^9^/L after 4 weeks of treatment, also showing a highly significant difference between the two (*t* = 8.252, *p* < 0.001). The Hs-CRP level before treatment was (9.73 ± 2.98) mg/L, which decreased to (3.46 ± 1.02) mg/L after 4 weeks of treatment, demonstrating a highly significant difference between the two (*t* = 25.02, *p* < 0.001) (Figure 2).

### Comparison of CXCL13, WBC, Hs-CRP levels in patients with stroke neurosyphilis after treatment

In evaluating the effectiveness of treatments for stroke neurosyphilis patients, outcomes varied with 58 cases (36.71%) showing significant effectiveness, 69 cases (43.67%) being effective, and 31 cases (19.62%) categorized as ineffective, resulting in an overall effectiveness rate of 80.38%. Analysis of CSF biomarkers prior to treatment revealed that the ineffective group (31 cases) had significantly higher mean CXCL13 levels of 133.91 ± 12.42 μg/mL compared to the effective group (111.18 ± 10.51 μg/mL), indicating a statistically significant difference (*t* = 17.56, *p* < 0.001). Similarly, the pre-treatment WBC count was higher in the ineffective group (5.48 ± 1.89*10^9^/L) versus the effective group (4.87 ± 1.57*10^9^/L), with this difference also being statistically significant (t = 3.121, *p* = 0.002). Additionally, pre-treatment high-sensitivity C-reactive protein (Hs-CRP) levels were higher in patients with ineffective outcomes, averaging 10.14 ± 3.12 mg/L compared to 8.56 ± 2.75 mg/L in those with effective outcomes, with a significant difference (*t* = 4.775, *p* < 0.001) observed.

### Univariate analysis of rehabilitation efficacy of HBO combined with TUS-NMES in stroke patients with neurosyphilis

No significant differences were observed between the ineffective and effective treatment groups in terms of age, sex, education level, smoking history, drinking history, presence of hypertension or diabetes, position of limb dysfunction, BMI, and initial antibody titers, with all *p*-values exceeding 0.05. However, notable differences were present in the pre-treatment scores of the FMA, MoCA, MBI, and levels of CXCL13, white blood cells (WBC), and Hs-CRP, all of which were statistically significant with p-values less than 0.05 (Table 3).

**Table 3:** Univariate analysis of rehabilitation efficacy of HBO combined with TUS-NMES in stroke patients with neurosyphilis.

Variables	Ineffective group (*n* = 31)	Effective group (*n* = 127)	t/χ2	P
Age (years)	50.29 ± 4.35	51.95 ± 4.43	1.877	0.062
Gender [n (%)]			3.427	0.064
Male	18 (58.06%)	95 (74.80%)		
Female	13 (41.94%)	32 (25.20%)		
Education level [n (%)]			3.583	0.166
Primary and middle school	12 (38.71%)	69 (54.33%)		
High school/technical school	9 (29.03%)	35 (27.56%)		
College and above	10 (32.26%)	23 (18.11%)		
Smoking history [n (%)]	15 (48.39%)	81 (63.78%)	2.476	0.116
Drinking history	19 (61.29%)	86 (67.72%)	0.462	0.497
Hypertension	12 (38.71%)	30 (23.62%)	2.906	0.088
Diabetes	9 (29.03%)	31 (24.41%)	0.282	0.596
Location of limb dysfunction [n (%)]			0.629	0.729
Left	14 (45.16%)	54 (42.52%)		
Right	14 (45.16%)	65 (51.18%)		
Both	3 (9.68%)	8 (6.30)		
BMI (kg/m^2^)	22.64 ± 2.61	22.48 ± 2.55	0.312	0.756
Pre-treatment FMA Score (points)	75.47 ± 4.47	82.91 ± 6.31	6.189	<0.001
Pre-treatment MoCA Score (points)	17.47 ± 2.11	20.91 ± 2.65	6.721	<0.001
Pre-treatment MBI Score (points)	60.42 ± 5.24	69.94 ± 6.50	7.57	<0.001
Pre-treatment CXCL13 Level (μg/mL)	133.91 ± 12.42	111.18 ± 10.51	17.56	<0.001
Pre-treatment WBC Level (10^9^/L)	5.48 ± 1.89	4.87 ± 1.57	3.121	0.002
Pre-treatment Hs-CRP Level (mg/L)	10.14 ± 3.12	8.56 ± 2.75	4.775	<0.001

### Multivariate analysis of rehabilitation efficacy of HBO combined with TUS-NMES in stroke patients with neurosyphilis

**Figure 3:**
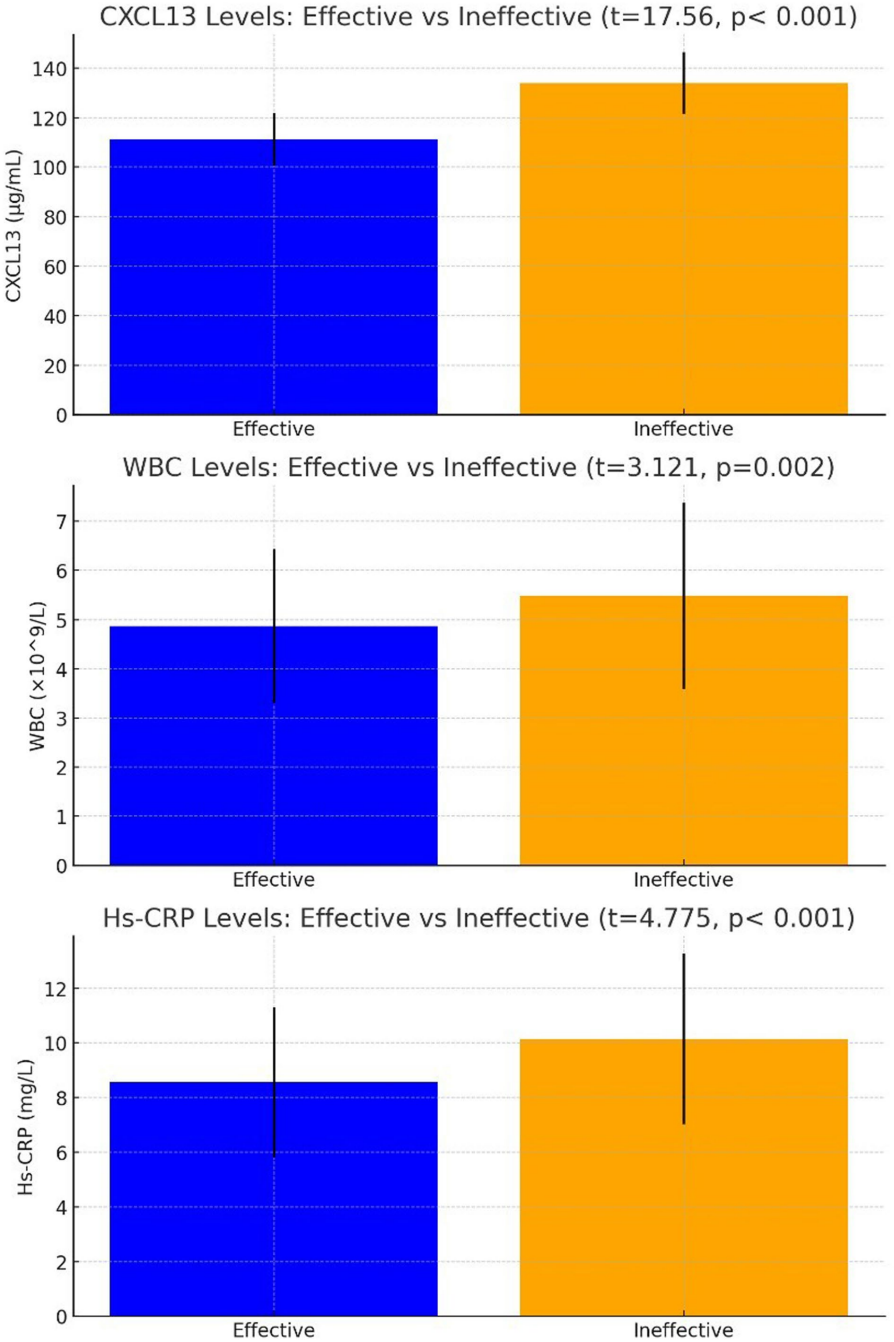
Comparison of CXCL13, WBC, Hs-CRP levels in patients with stroke neurosyphilis after treatment.

In the multivariate analysis of variance, which included Fugl-Meyer Assessment (FMA), Montreal Cognitive Assessment (MoCA), Modified Barthel Index (MBI), CXCL13, white blood cell (WBC), and high-sensitivity C-reactive protein (Hs-CRP), significant predictors of treatment effectiveness were identified. The levels of CXCL13 in cerebrospinal fluid before treatment were significantly associated with treatment outcomes (*p* = 0.013), with an odds ratio (OR) of 2.875 and a 95% confidence interval (CI) ranging from 1.252 to 6.600. Similarly, pre-treatment WBC levels showed a significant influence (*p* = 0.042, OR not provided, 95%CI = 1.041–9.058), as did the levels of Hs-CRP (*p* = 0.017, OR = 4.237, 95%CI = 1.537–11.682) (Table 4), underscore the predictive value of these biomarkers in determining the effectiveness of the treatment regimen for stroke neurosyphilis patients (Figure 3).

**Table 4:** Multivariate analysis of rehabilitation efficacy of HBO combined with TUS-NMES in stroke patients with neurosyphilis.

	Estimated value	Standard error	Wald Chi square	*P*	OR	95% CI
FMA score before treatment	1.042	0.741	1.977	0.16	2.835	0.663–12.144
MoCA score before treatment	0.984	0.654	2.264	0.132	2.675	0.742–9.639
MBI score before treatment	1.273	0.691	3.394	0.065	3.572	0.922–13.837
CXCL13	1.056	0.424	6.203	0.013	2.875	1.252–6.600
WBC	1.125	0.522	4.123	0.042	3.071	1.041–9.058
Hs-CRP	1.347	0.587	5.763	0.017	4.237	1.537–11.682

### ROC curve of single index and multi-index combination in neurosyphilis

**Figure 4:**
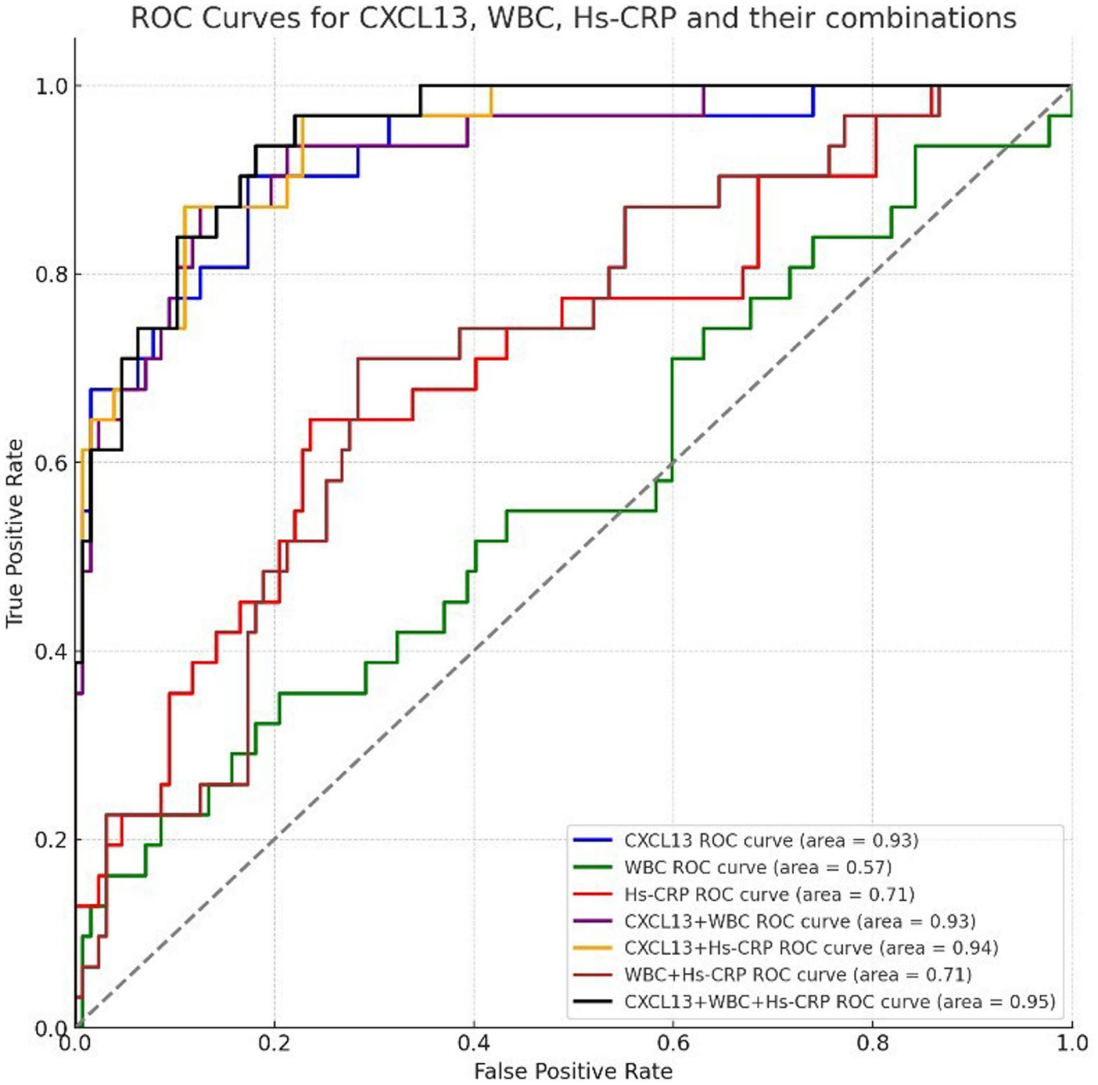
ROC curve of single index and multi-index combination in neurosyphilis.

The receiver operating characteristic (ROC) curve analysis revealed differing predictive capabilities for the biomarkers CXCL13, WBC, and Hs-CRP in assessing the rehabilitation outcomes of stroke patients with neurosyphilis. Specifically, the area under the curve (AUC) for CXCL13 was 0.908, indicating a strong predictive ability. In contrast, WBC and Hs-CRP yielded lower AUC values of 0.582 and 0.659, respectively, suggesting these markers have limited discriminatory power when used individually (Figure 4).

When analyzing combinations of these indicators, the AUC values improved significantly, indicating enhanced predictive accuracy. The combinations of CXCL13 + WBC, CXCL13 + Hs-CRP, and WBC + Hs-CRP yielded AUCs of 0.947, 0.953, and 0.693, respectively. Notably, the combination of all three biomarkers, CXCL13 + WBC + Hs-CRP, provided the highest predictive value with an AUC of 0.958. These findings, illustrated in Figure 4, underscore the advantage of using a composite biomarker approach over single indicators for predicting rehabilitation outcomes in this patient population (Figure 4).

## Discussion

Early neurosyphilis often initiates a vascular inflammatory pathway that can lead to intracranial vascular stenosis, occlusion, and thrombosis, culminating in acute ischemic stroke. This progression frequently results in significant damage to the central nervous system and limb dysfunction, thereby extending hospital stays, increasing disability rates, and imposing a substantial burden on families and society (3, 14). Beyond merely eradicating *Treponema pallidum*, it is crucial to identify treatments that are safe, effective, and economical to alleviate the somatic symptoms experienced by these patients. Currently, various rehabilitation schemes exist for addressing the aftermath of acute ischemic stroke; however, their efficacy in managing stroke neurosyphilis specifically has yet to be fully established. Consequently, this study was conducted to assess the effectiveness of a combined HBO and TUS-NMES rehabilitation therapy for this patient population.

The study’s findings revealed that after 4 weeks of treatment, there were notable improvements in the FMA, MoCA, and MBI scores. This suggests that the combined use of HBO and TUS-NMES can effectively enhance limb function, cognitive function, and self-care ability in patients with stroke neurosyphilis, indicating a promising rehabilitation treatment effect. The potential mechanisms behind these improvement include HBO’s capacity to enhance oxygenation of brain cells and improve microcirculation within brain tissues, while also inducing systemic vascular contraction under hyperbaric conditions. This process selectively reduces blood flow in the internal carotid artery but increases it in the vertebral artery and brain stem, ensuring that the reticular structure of the brain receives ample oxygen, thus supporting effective patient outcomes (15, 16).

Further studies highlight that stroke neurosyphilis patients frequently experience nerve injuries, limb dysfunction, and cortical motor area dysfunction resulting from abnormal neuronal activity in the cerebral hemispheres, which leads to impaired motor control (17). The TUS-NMES treatment leverages ultrasound to stimulate the recovery of the cerebral hemisphere cortex by targeting the brain lesion sites, which impacts the deep brain structures and aids in nerve repair. Additionally, NMES targets critical areas of limb dysfunction, where repeated muscle stimulation helps restore local blood flow and rebuild lost neuromuscular functions (18, 19). Therefore, the integration of HBO and TUS-NMES not only addresses limb and cognitive dysfunctions but also enhances the overall efficacy of rehabilitation treatments for patients with stroke neurosyphilis.

CXCL13, a chemokine ligand, is notably elevated in the CSF of neurosyphilis patients compared to those without the condition. When *Treponema pallidum* first invades the CNS, it triggers a robust immune response characterized by the recruitment of B lymphocytes and plasma cells into the CSF. This, in turn, drives the upregulation of CXCL13—an integral chemokine in B-cell chemotaxis. *Treponema pallidum*’s invasion leads to an inflammatory response characterized by the accumulation of B cells in the CSF, abnormally high CXCL13 expression, and subsequent white blood cell infiltration. These processes contribute to endothelial cell apoptosis and impair neurological functions (20–22). In the absence of specific treatment, persistently elevated CXCL13 levels can be detected for extended periods—often months or even years—reflecting ongoing inflammation and continued pathogen activity within the CNS (23, 24). Over time, this sustained inflammatory milieu can lead to progressive neural tissue damage and a higher likelihood of complications such as vascular inflammation and stroke. Conversely, in patients receiving adequate antisyphilitic therapy, CXCL13 levels typically begin to decline within weeks to months as *Treponema pallidum* is eradicated and the intrathecal inflammatory response subsides (24, 25). However, the rate of decrease can vary among individuals due to factors such as disease stage, immune status, and treatment adherence. Some studies suggest that although CXCL13 levels may drop substantially post-treatment, mild elevations can persist for a period of time even after clinically effective therapy, possibly reflecting residual immune activation or incomplete clearance of inflammatory debris (26). These temporal dynamics highlight the potential utility of CXCL13 measurement not only as a diagnostic marker but also as an indicator of therapeutic response over time. In our study, we observed a significant reduction in CXCL13 levels after 4 weeks of adjunct therapy with HBO and TUS-NMES—an improvement that may be partly attributable to better CNS perfusion, inflammation control, and enhanced antibiotic efficacy. This finding underlines the value of monitoring CXCL13 in gauging both the activity of the disease and the effectiveness of the combined rehabilitation approach in stroke neurosyphilis.

Furthermore, CSF white blood cell count serves as a crucial diagnostic and assessment tool for neurosyphilis, a disease caused by the penetration of *Treponema pallidum* into the central nervous system, often manifesting in late-stage untreated syphilis patients. Additionally, Hs-CRP is employed to assess systemic inflammation and evaluate disease activity within neurosyphilis patients. After 4 weeks of treatment, notable reductions in CXCL13, CSF white blood cell count, and Hs-CRP levels are observed. HBO plays a role in regulating oxidative stress markers, reducing neutrophil chemotaxis, and controlling inflammation in the brain, thereby mitigating vasculitis caused by *Treponema pallidum*. Moreover, the ultrasound component of TUS-NMES promotes capillary network recovery, enhances neuronal activity, and positively affects CXCL13, white blood cell count, and Hs-CRP levels (27, 28).

This study systematically assessed the predictive accuracy of single and combined indicators—CXCL13, WBC, and Hs-CRP—for the rehabilitation outcomes of stroke patients with neurosyphilis by evaluating the AUC. The results demonstrated that CXCL13, an established inflammatory marker, had strong predictive capabilities, reflecting its significant association with both the inflammatory response and disease prognosis. In contrast, the AUC values for WBC and Hs-CRP were comparatively lower, suggesting that these markers alone might not provide sufficient predictive accuracy due to their susceptibility to various influencing factors.

The predictive performance significantly enhanced when combining multiple indicators. The AUC values for CXCL13 + WBC, CXCL13 + Hs-CRP, and WBC + Hs-CRP were 0.947, 0.953, and 0.693, respectively, highlighting that a multi-biomarker approach can yield more accurate predictions. The combination of all three indicators—CXCL13 + WBC + Hs-CRP—achieved the highest AUC value of 0.958, indicating the best predictive ability. This suggests that there are complementary effects among these biomarkers, which contribute to a more comprehensive and accurate prediction of patient outcomes.

These findings further highlight the substantial clinical value of CXCL13 and its combinations as predictors in neurosyphilis management. The relationship between neuronal activity and vasodilation significantly influences the velocity of CSF dynamics. TUS-NMES can directly target brain lesions, facilitate brain signal transmission and feedback, and positively affect the cerebral cortex to expedite the recovery of injured cells (29).

The metabolic and functional state of the cerebral cortex is intricately linked to CSF. When *Treponema pallidum* invades the CSF, CXCL13 levels rise. Under the influence of TUS-NMES, improvements in the cerebral cortex re-establish the microcirculation needed for brain metabolism, leading to controlled levels of CXCL13 (30). Consequently, monitoring changes in CXCL13 levels serves as a reliable index to evaluate the therapeutic effectiveness in stroke patients with neurosyphilis.

Furthermore, CXCL13 directly reflects the immune-inflammatory response following nervous system infection. The synergistic action of multiple cytokines within this immune response not only contributes to the development of acute ischemic stroke but also exacerbates neuronal damage (31). This underlines the importance of CXCL13 as both a standalone and combinatory biomarker in predicting and managing neurosyphilis, emphasizing its potential for guiding therapeutic strategies and improving patient outcomes.

Indeed, while the findings from this study underscore the robust predictive capacity of CXCL13, particularly when used in conjunction with WBC and Hs-CRP, they also highlight several avenues for further investigation to enhance the generalizability and efficacy of these biomarkers in clinical practice. Firstly, the predictive reliability of CXCL13 across diverse patient demographics remains to be established. The variability in immune system responses and underlying health conditions across different patient groups may influence the biomarker’s effectiveness, necessitating broader validation studies. Secondly, there is significant potential for identifying and integrating additional biomarkers into the existing predictive framework. By exploring a wider array of biomarkers, researchers could develop a more nuanced and potentially more accurate predictive model that accounts for various pathophysiological facets of neurosyphilis in stroke patients. Moreover, the predictive capabilities of these combined biomarkers, particularly in terms of their synergistic effects, should be tested in a larger cohort. This would not only bolster the confidence in using these biomarkers clinically but also refine the predictive models to ensure they are robust across various clinical settings and patient conditions. While our study does not include a control group, the effectiveness of HBOT and TUS-NMES in enhancing patient outcomes was evaluated through comparative baseline and post-treatment assessments within the same cohort. By analyzing patient conditions before and after the intervention, we have been able to document changes and improvements that can be attributed to the applied therapies. This within-subject design minimizes variability between different patient groups and provides a clear timeline of recovery linked to the interventions. Nevertheless, we recognize that this method cannot completely rule out the effects of standard treatments such as penicillin or general stroke management. Therefore, while our results suggest significant improvements associated with the additional therapies, conclusions about their exclusive effects should be interpreted with caution. To overcome this limitation and definitively separate the contributions of each treatment, future studies should aim to incorporate a randomized controlled design with a distinct control group receiving only standard care.

In conclusion, this study reinforces the critical role of a multi-biomarker approach in enhancing the accuracy of predicting rehabilitation outcomes in stroke patients with neurosyphilis. The combined use of CXCL13, WBC, and Hs-CRP, in particular, presents a valuable tool for clinicians. This promising avenue of research provides a solid foundation for further refinement and optimization of therapeutic strategies, potentially leading to improved patient care and outcomes.

## Data Availability

The raw data supporting the conclusions of this article will be made available by the authors, without undue reservation.
